# Critically Ill COVID-19 Patients Exhibit Anti-SARS-CoV-2 Serological Responses

**DOI:** 10.3390/pathophysiology28020014

**Published:** 2021-05-17

**Authors:** Douglas D. Fraser, Gediminas Cepinskas, Marat Slessarev, Claudio M. Martin, Mark Daley, Maitray A. Patel, Michael R. Miller, Eric K. Patterson, David B. O’Gorman, Sean E. Gill, Ian Higgins, Julius P. P. John, Christopher Melo, Lylia Nini, Xiaoqin Wang, Johannes Zeidler, Jorge A. Cruz-Aguado

**Affiliations:** 1 Lawson Health Research Institute, London, ON N6C 2R5, Canada; gcepinsk@uwo.ca (G.C.); Marat.Slessarev@lhsc.on.ca (M.S.); cmartin1@uwo.ca (C.M.M.); mdaley2@uwo.ca (M.D.); Michael.Miller@lhsc.on.ca (M.R.M.); Eric.Patterson@lhsc.on.ca (E.K.P.); dogorman@uwo.ca (D.B.O.); sgill8@uwo.ca (S.E.G.); 2 Department of Pediatrics, Western University, London, ON N6A 3K7, Canada; 3 Department of Clinical Neurological Sciences, Western University, London, ON N6A 3K7, Canada; 4 Department of Physiology & Pharmacology, Western University, London, ON N6A 3K7, Canada; 5 Department of Medical Biophysics, Western University, London, ON N6A 3K7, Canada; 6 Department of Medicine, Western University, London, ON N6A 3K7, Canada; 7 Department of Computer Science, Western University, London, ON N6A 3K7, Canada; mpate267@uwo.ca; 8 The Vector Institute for Artificial Intelligence, Toronto, ON M5G 1M1, Canada; 9 Department of Surgery, Western University, London, ON N6A 3K7, Canada; 10 Department of Biochemistry, Western University, London, ON N6A 3K7, Canada; 11 Diagnostics Biochem Canada, London, ON N6M 1A1, Canada; ihiggins@dbc-labs.com (I.H.); juliuspaul78@gmail.com (J.P.P.J.); cmelo@dbc-labs.com (C.M.); lnini@dbc-labs.com (L.N.); cwang@dbc-labs.com (X.W.); jzeidler@dbc-labs.com (J.Z.); jcruz@dbc-labs.com (J.A.C.-A.)

**Keywords:** COVID-19, intensive care unit, humoral response, serology, immunoglobulins, outcome

## Abstract

Coronavirus disease 2019 (COVID-19), caused by SARS-CoV-2, is a global health care emergency. Anti-SARS-CoV-2 serological profiling of critically ill COVID-19 patients was performed to determine their humoral response. Blood was collected from critically ill ICU patients, either COVID-19 positive (+) or COVID-19 negative (−), to measure anti-SARS-CoV-2 immunoglobulins: IgM; IgA; IgG; and Total Ig (combined IgM/IgA/IgG). Cohorts were similar, with the exception that COVID-19+ patients had a greater body mass indexes, developed bilateral pneumonias more frequently and suffered increased hypoxia when compared to COVID-19- patients (*p* < 0.05). The mortality rate for COVID-19+ patients was 50%. COVID-19 status could be determined by anti-SARS-CoV-2 serological responses with excellent classification accuracies on ICU day 1 (89%); ICU day 3 (96%); and ICU days 7 and 10 (100%). The importance of each Ig isotype for determining COVID-19 status on combined ICU days 1 and 3 was: Total Ig, 43%; IgM, 27%; IgA, 24% and IgG, 6%. Peak serological responses for each Ig isotype occurred on different ICU days (IgM day 13 > IgA day 17 > IgG persistently increased), with the Total Ig peaking at approximately ICU day 18. Those COVID-19+ patients who died had earlier or similar peaks in IgA and Total Ig in their ICU stay when compared to patients who survived (*p* < 0.005). Critically ill COVID-19 patients exhibit anti-SARS-CoV-2 serological responses, including those COVID-19 patients who ultimately died, suggesting that blunted serological responses did not contribute to mortality. Serological profiling of critically ill COVID-19 patients may aid disease surveillance, patient cohorting and help guide antibody therapies such as convalescent plasma.

## 1. Introduction

Coronavirus disease 2019 (COVID-19) is caused by SARS-CoV-2. Critically ill COVID-19 patients are admitted to the intensive care unit (ICU), where the mortality rate is approximately 42% [1]. While a variety of patient risk factors have been identified, the patient and infection attributes that contribute to ICU mortality are generally unknown. Once infected, the body responds with the innate immune response [2,3]. An exaggerated innate response has been suggested to underlie severe COVID-19 disease, referred to as a ‘cytokine storm’, with evidence for increased interferons, TNF, bradykinin and serine proteases [4,5,6]. Poorer outcomes have also been attributed to microvascular disease [7], which is associated with microthrombi [8].

The innate reaction is followed by a humoral immune response, with production of antigen-specific antibodies, or immunoglobulins (Ig) [9]. Five antibody isotypes are named alphabetically based on their heavy chain class: alpha (IgA), delta (IgD), epsilon (IgE), gamma (IgG), and mu (IgM). The intensity and duration of the SARS-CoV-2 humoral response has been partially investigated [10,11], with studies demonstrating that the response rate and the time to seroconversion are both variable depending on the targeted antigen, the Ig isotype investigated, and the assay platform used [12]. The IgM response is detected by 6–14 days after infection, while an IgG response begins shortly thereafter [13]. Serological studies in critically ill patients are few and limited by insufficient sampling time points and/or focus on only one immunoglobulin isotype [14,15].

Serological profiling of critically ill COVID-19 patients over their ICU stay was the overall aim of this study. Our specific objectives were: (1) to determine and compare the serological responses between COVID-19-positive (+) ICU patients and either healthy control subjects or COVID-19-negative (−) ICU patients; (2) to determine which Ig isotypes dominate the serological responses in COVID-19+ patients; and (3) to determine whether the serological responses differ between COVID-19+ patient outcome.

## 2. Results

We investigated and compared 4 age- and sex-matched populations: 14 critically ill COVID-19+ patients (median years of age = 61.0, IQR = 54.0, 67.0), 14 critically ill COVID-19- patients (median years of age = 58.5, IQR = 52.5, 63.0), 14 mildly ill non-hospitalized COVID-19+ patients (median years of age = 60.0, IQR = 55.8, 65.0), and 14 healthy controls (median years of age = 57.5, IQR = 53.3, 63.0; *p* = 0.645). Baseline demographic characteristics, comorbidities, laboratory values, and chest x-ray results are reported in Table 1. The COVID-19+ patients had a higher body mass index and developed bilateral pneumonia more frequently, while COVID-19- patients were more likely to suffer unilateral pneumonia. COVID-19+ patients had lower PaO_2_:FiO_2_ ratios when compared to COVID-19- patients, and were more likely to receive high-flow oxygen therapy. Sepsis was ‘confirmed’ by infectious pathogen identification in only 28.6% of COVID-19- patients, with sepsis ‘suspected’ in the remaining 71.4%. The mortality rate was 50% for COVID-19+ patients.

Serum levels of immunoglobulins (IgM, IgA, IgG and Total Ig) were measured using previously validated immunoassays (Table 2), which performed well when compared to other commercially available serology assays [16]. The only exception was the anti-SARS-CoV-2 IgA ELISA that reported a positive percent agreement (PPA) of 85.7%, which may reflect the brief period of IgA antibody prevalence that is highly variable between individuals. Additionally, some individuals are IgA-deficient and therefore, IgA antibodies are not produced upon a respiratory infection [17]. The precision of the immunoassays used in the study was in line with the precision typically generated by ELISAs when all the sources of variation are included in the analysis.

**Figure 1:**
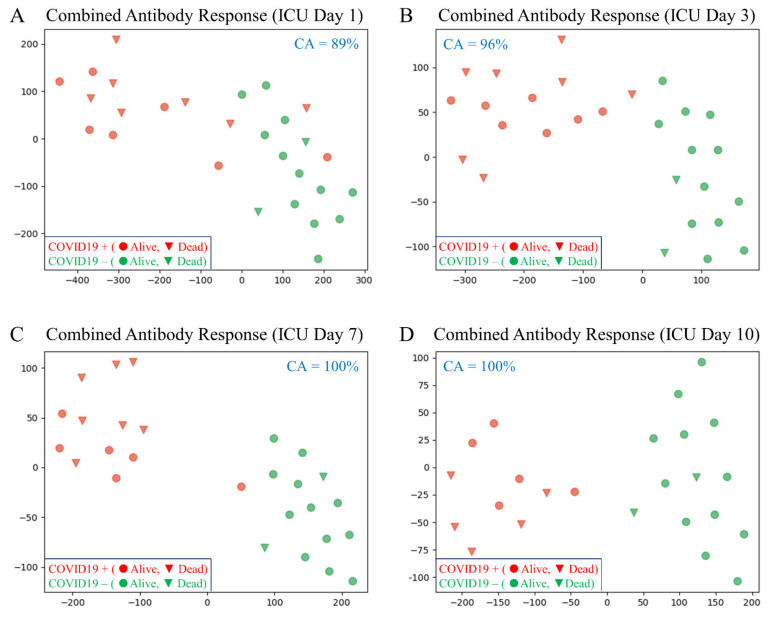
T-distributed stochastic neighbor embedding (tSNE) plots comparing the integrated combined antibody responses (IgM, IgA and IgG) between COVID-19+ and COVID-19- patients on different ICU days (the axes are dimension-less). In all plots, subjects are indicated in two dimensions following a dimensionality reduction in their respective antibody responses (the classification accuracy [CA] is indicated for each comparison). Green dots represent COVID-19- patients, while red dots represent age- and sex-matched COVID-19+ patients. The clinical outcome for all patients is shown as alive (circle) or dead (triangle). (**A**) ICU day-1; CA = 89%). The dimensionality reduction shows some mixing of COVID-19+ patients with COVID-19- patients, suggesting that not all COVID-19+ patients have developed a significant antibody response on ICU day-1. (**B**) The dimensionality reduction shows minimal mixing of COVID-19+ patients with COVID-19- patients, suggesting that most COVID-19+ patients have developed a significant antibody response by ICU day-3 (CA = 96%). (**C**,**D**) The dimensionality reduction shows that the two cohorts are distinct and easily separable with all COVID-19+ patients having developed robust antibody responses by ICU days -7 and -10 (CA = 100%).

Figure 1 shows four t-distributed stochastic neighbor embedding (t-SNE) plots corresponding to the combined antibody response on four separate ICU days (integration of IgM, IgA and IgG). The combined antibody response can distinguish COVID-19+ versus COVID-19- patients with increasing accuracy: ICU day-1, 89%; ICU day-3, 96%; ICU day-7, 100% and ICU day-10, 100%. Feature ranking provided the importance of each Ig assay for determining COVID-19 status on combined ICU days-1 and -3 (Total Ig, 43%; IgM, 27%; IgA, 24% and IgG, 6%).

Only 21% (*n* = 3/14) of critically ill COVID-19+ patients failed to exhibit a serological response on ICU day-1; however, these 3 COVID-19+ patients developed Ig responses by ICU day 3. In contrast, all COVID-19- patients (*n* = 14) fell below the established cut-off values and, therefore, were considered serologically negative. All healthy control subjects (*n* = 14) fell below the established cut-off values with the exception of one subject who exhibited a weak IgM response, suggesting mild cross reactivity to another antigen in this one healthy control subject (sample collected prior to November 2019).

We also compared the Ig responses between critically ill (ICU day-3 or near maximal response) and mildly ill non-hospitalized COVID-19+ patients. Both cohorts displayed similar Ig responses despite differences in disease severity (all Ig comparisons non-significant; Table 3).

**Figure 2:**
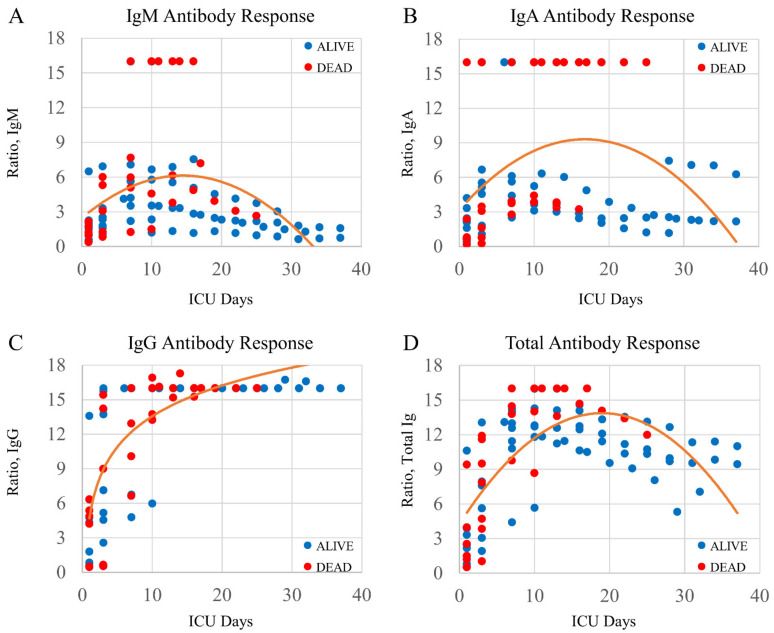
Plots demonstrating Ig isotype responses in COVID-19+ patients over multiple ICU days. Four plots with a best-fit line through the data points. Each Ig isotype has a unique time course peak: (**A**) IgM transiently peaking on or about ICU day-13; (**B**) IgA transiently peaking on or about ICU day-17; and (**C**) IgG persistently increasing. (**D**) The total antibody assay shows an Ig peak on approximately ICU day-19. The clinical outcome for all patients is shown as alive (blue circle) or dead (red circle).

Figure 2 shows the Ig responses for all COVID-19 patients (*n* = 14). Each data set is overlaid with a best fit curve. The peak responses for each antibody subgroup occurred temporally in the following order: IgM on ICU day-13; IgA on ICU day-17; and IgG that persistently increased. The peak response in the Total Ig occurred on approximately ICU day 19.

The Ig responses between COVID-19 patients that either survived to ICU discharge or expired in the ICU were evaluated using two approaches. We first examined whether Ig concentrations were different on ICU day-1 between critically ill COVID-19+ patients that lived or died; there were no significant differences identified (IgM, *p* = 0.749; IgA, *p* = 0.277. IgG, *p* = 0.522, Total Ig, *p* = 0.949). We then examined if Ig concentrations were different in critically ill COVID-19+ patients that lived or died over their entire ICU stay. There were significant interactions between days after ICU admission and COVID-19 patient outcome (alive versus dead) for IgA (*F*(1,93) = 13.27, *p* < 0.001, η^2^ = 0.13) and for Total Ig (*F*(1,93) = 9.20, *p* = 0.003, η^2^ = 0.09), indicating that patients who died had higher IgA and Total Ig levels earlier in their ICU stay compared to patients who survived. In contrast, there were no significant interactions between COVID-19 patient outcomes and either IgM (*p* = 0.223) or IgG (*p* = 0.053).

## 3. Discussion

In this study, we utilized 4 validated SARS-CoV-2 antibody immunoassays to measure Ig isotypes in serum obtained from 4 age- and sex-matched populations: 14 critically ill COVID-19+ patients, 14 critically ill COVID-19- patients, 14 mildly ill non-hospitalized COVID-19+ patients, and 14 healthy controls. Given the number of Ig isotypes measured, we analyzed the data with state-of-the-art machine learning, as well as conventional statistics. Our data indicate the presence of a robust COVID-19 serological response that was evident in all COVID-19 patients by ICU day-7. Moreover, the expected temporal patterns were present for all individual Ig isotypes with peak responses occurring in the following order: IgM on ICU day-13 > IgA on ICU day-17 > IgG that persistently increased/plateaued. Finally, we determined that COVID-19 patients who died not only had an equally robust serological response to those COVID-19 patients who survived, but they also showed an earlier rise in IgA and Total Ig, the last likely due to the contribution of IgA to the total antibody response. Our exploratory data suggest that critically ill COVID-19 patients develop robust serological responses that might not protect from poor outcome. 

Our COVID-19+ ICU patients were comparable to those reported in other studies [18,19,20,21] with respect to demographic, comorbidities and clinical presentation. In contrast to COVID-19- ICU patients, our COVID-19+ ICU patients developed bilateral pneumonia more frequently and they had lower PaO_2_/FiO_2_ ratios. A unique COVID-19 inflammatory profile was previously characterized in many of these same patients and showed elevated interferons, TNF and serine proteases [5,6], and a thrombotic profile associated with endothelial activation and glycocalyx degradation [7]. We also employed targeted proteomics and metabolomics, thereby identifying novel biomarkers that accurately predict COVID-19 poor outcome [22,23]. Taken together, COVID-19 is a severe illness with a unique pathophysiological signature, as well as a high mortality rate. Despite standardized ICU care, mortality in critically ill COVID-19 patients was 50%.

In all four SARS-CoV-2 Ig assays used here, a ROC analysis yielded an area under the curve (AUC) higher than 0.987, indicating an outstanding discrimination between positive and negative samples, a performance that persisted in studies including blood serum and plasma specimens from nearly 300 individuals presenting an elevated titer of antibodies against a broad spectrum of other infectious diseases (Table 2). In fact, the assays used in this study performed well when compared to other commercially available serological assays [16]. The viral S1 spike protein region used as antigen is currently a target of several vaccines against SARS-CoV-2, which might potentially expand the applications of these assays to measure vaccination seroconversion rates.

Our data showed that all critically ill COVID-19+ patients demonstrated a characteristic serological response on or before ICU day-7, as did all COVID-19+ subjects with mild symptoms not requiring hospitalization. With regard to critically ill COVID-19+ patients, the variability in antibody response may reflect the degree of viral load and/or delays between infection and symptom onset requiring ICU admission. In contrast, none of the age- and sex-matched COVID-19- ICU patients had a measurable serological response. In the critically ill COVID-19+ patients, the temporal peak for each Ig isotype was as expected for Ig isotypes [12,13], with approximate average peaks for IgM and IgA on ICU days-13 and -17, respectively. Serum IgG continued to increase to a steady state over the ICU course, while Total Ig peaked on ICU day-19. Published data indicate a 38.3% antibody response during the first 7 days of SARS-CoV-2 infection, and an 89.6% antibody response during the second week of infection [11], consistent with the temporal course described in this study. Others have demonstrated that 100% of COVID-19 patients expressed IgG by 17–19 days after onset of their symptoms [10].

The magnitude of SARS-CoV-2 antibody production after infection was similar between COVID-19 patients with variable disease severity, as we have also shown here, before post-infectious day 12; however, antibody levels diverged to reflect disease severity by 14 days after infection [11]. Our data demonstrate similar serological responses in COVID-19 ICU patients who either died or survived. Moreover, those COVID-19 patients who died had a faster rate of rise in IgA and Total Ig. Taken together, our data suggest that a blunted serological response does not contribute to COVID-19 mortality, and that the faster antibody rise associated with COVID-19 mortality may reflect increased viral load [24]. SARS-CoV-2 load was associated with both greater cytokine production and lung injury [25], suggesting that cytokine modulators may be of therapeutic benefit, such as TNF inhibitors [6]. 

Currently, there are no targeted therapies for COVID-19. Thus, there is interest in human convalescent serum as a therapeutic option for prevention and treatment of COVID-19 [26]. Ig containing serum requires sufficient numbers of people who have recovered from COVID-19 and can donate blood. While historical evidence suggests that IgG administration may prevent disease (i.e., respiratory syncytial virus), treatment of active COVID-19 disease is controversial due to mixed results in clinical trials [27,28]. Given that the potential risks of convalescent serum administration include a transfusion reaction and/or blunting of the endogenous humoral response, a personalized medicine approach is warranted [29]. Indeed, profiling of anti-SARS-CoV-2 Ig (particularly IgG), as we have done here, may be useful when combined with other clinical, genetic [30] and lifestyle [31] factors to determine patient candidates that will benefit from this therapy. 

Our serological profiling in critically ill COVID-19 patients was novel, but also had several limitations. First, due to patient sampling methodologies we cannot determine the disease onset or quantify symptom duration; however, ICU admission has strict criteria for advanced monitoring or interventions and serves as a reasonable surrogate. Second, while our study identified serological responses that failed to protect patients from a poor outcome, our overall COVID-19 study population was limited in number and highlights the need for larger cohort patient studies. Third, we report only mortality as our primary clinical outcome. Fourth, due to the limited number of enrolled patients, our data must be interpreted with caution. Future studies with larger sample sizes can explore whether serological responses correlate with additional clinical outcomes such as functional status in survivors. Finally, our analyses employed a cross-validation methodology, which is a standard, accepted technique in machine learning, but should be validated on a larger testing set that is used only once. Overfitting was minimized by using a very small number of trees, and the very limited depth was protective against over-fitting [32].

## 4. Conclusions

In summary, we report serological responses in COVID-19 ICU patients, with expected temporal responses for individual Ig isotypes. While exploratory, our study filled a knowledge gap on the intensity and pattern of humoral responses expressed by critically ill COVID-19 patients. Given the rapid spread of COVID-19 and the critical need for therapies, our data may be important for refining future clinical trials with anti-SARS-CoV-2 antibody infusion.

## 5. Materials and Methods

### 5.1. Study Participants and Clinical Data

We enrolled consecutive patients who were admitted to our level-3 academic ICUs at London Health Sciences Centre (London, ON, Canada) and were suspected of having COVID-19 based on standard hospital screening procedures [33]. Blood sampling began on ICU admission for up to 3 days in COVID-19- patients, or up to 7 days in COVID-19+ patients followed by every 3 days until death or discharge. COVID-19 status was confirmed as part of standard hospital testing by detection of two SARS-CoV-2 viral genes using polymerase chain reaction (Roche SARS-CoV-2 PCR kits, Roche Diagnostics, Rotkreuz, Switzerland; FDA Authorized) [34]. Patient baseline characteristics were recorded on ICU admission and interventions were documented [5,6,7,15,22,23]. Disease severity scores were calculated [35,36]. Both patient groups were characterized as having confirmed or suspected sepsis diagnosis using Sepsis 3.0 criteria [36]. For serological comparisons, we included both non-hospitalized COVID-19+ patients with mild disease, and healthy control subjects who were public volunteers without disease, acute illness or prescription medications (https://translationalresearchcentre.com/; accessed on 13 May 2021) [37,38]. Final participant groups were constructed by age and sex matching.

### 5.2. Blood Draws

Standard operating procedures were used to ensure all samples were treated rapidly and equally [5,6,7,15,22,23,39]. Daily blood was obtained from critically ill ICU patients via indwelling catheters using vacuum serum separator tubes and placed immediately on ice. If a venipuncture was required, research blood draws were coordinated with a clinically indicated blood draw. In keeping with accepted research phlebotomy protocols for adult patients, blood draws did not exceed maximal volumes [40]. Once transferred to a negative pressure hood, blood was centrifuged and sera isolated, aliquoted at 250 µL and frozen at −80 °C. All samples remained frozen until use and freeze/thaw cycles were avoided.

### 5.3. Immunoassays

Anti-SARS-CoV-2 antibodies (IgG, IgM, IgA, and Total Ig) were detected with four separate enzyme-linked immunosorbent assays (ELISAs) kits developed at Diagnostics Biochem Canada Inc. (https://dbc-labs.com/, accessed on 13 May 2021; London, ON, Canada; Table 2). The antigen used in the anti-SARS-CoV-2 serology ELISA tests is a recombinant spike protein S1 subunit, RBD domain (aa 319–541). The “Total Ig” test kit detected the sum of all three Ig isotypes (IgG, IgM and IgA). The tests were performed in accordance with the ‘information for use’ (IFU) provided with each kit. Briefly, all four serological tests were based on a sandwich immunoassay configuration. The antigen, coated to the microplate wells, comprises one of the most specific protein regions of the SARS-CoV-2 virus enabling low cross-reactivity with other antibodies generated by numerous other viruses, including other human coronaviruses. Anti-SARS-CoV-2 antibodies in serum that bind to the antigen were detected with isotype-specific horse-radish peroxidase-conjugated antibodies (for IgG, IgM and IgA tests) or with a mix for the Total Ig kit. Cut-off values had been determined independently for each test during the design phase with a receiver operating characteristic (ROC) analysis-based classification of samples previously known to be SARS-CoV-2 positive or negative. The ratio between the serum sample optical density (OD) and the OD of the negative control, adjusted with a calibration factor to match the cut-off, was used to calculate the serum “ratio”. Sera with a ratio higher than the cut-off were considered positive. 

The immunoassay kit’s precision was evaluated according to the Clinical and Laboratory Standards Institute (CLSI) guideline EP5-A3 (Table 2; provided by Diagnostics Biochem Canada Inc, London, ON, Canada; https://dbc-labs.com/, accessed 13 May 2021). The clinical sensitivity of each immunoassay (positive percent agreement, PPA) was validated with samples that tested positive for SARS-CoV-2 by PCR (for IgG and the total antibodies ELISAs) or both for PCR and a prior immunoassay test (for IgA and IgM ELISAs). The clinical specificity (negative percent agreement, NPA) was validated against samples collected before the COVID-19 pandemic (November 2019) and, therefore, considered SARS-CoV-2 negative. The overall percent agreement (OPA) was calculated as the total number of times in which the immunoassays agreed with the pre-analytical status of the specimen (positive or negative) divided by the total number of readings.

### 5.4. Population Statistics

Medians (IQRs) and frequency (%) were used to report ICU patient baseline characteristics for continuous and categorical variables, respectively; continuous variables were compared using Mann–Whitney U tests (or Kruskal–Wallis tests, as appropriate), and categorical variables were compared using Fisher’s exact chi-square. Linear, quadratic, or logarithmic (as appropriate for best model fit) regression analyses were conducted separately for each antibody in order to examine the interaction between ICU days over time and those patients who lived or died. All population statistics were conducted using SPSS version 26 (IBM Corp., Armonk, NY, USA). *p*-values < 0.05 were considered significant.

### 5.5. Machine Learning

COVID-19 analyte data were visualized with a non-linear dimensionality reduction on the full data matrix using the t-distributed stochastic nearest neighbor embedding (t-SNE) algorithm [41]. t-SNE attempts to find an optimal non-linear projection of the observed data onto a manifold with complex geometry, but low dimension, embedded in the full dimensional space of the raw data. A random forest classifier was also trained on the variables to predict either COVID-19 status or COVID-19 outcome. A random forest is a set of decision trees that can be interrogated to identify the features that have the highest predictive value. To control overfitting, COVID-19 status was determined using a seven-fold cross validation with a random forest of ten trees with a max depth of three [32]. Data from ICU day 3 COVID-19- patients were used as a proxy for day 7 and day 10 COVID-19- patients when conducting the analysis.

## Figures and Tables

**Table 1:** Demographics and clinical data for all age- and sex-matched study subjects.

Variable	COVID-19+ Critically Ill	COVID-19− Critically Ill	COVID-19+ Mildly Ill	Healthy Controls	*p*-Value
*n*	14	14	14	14	1.000
Age in years	61.0 (54.0, 67.0)	58.5 (52.5, 63.0)	60.0 (55.8, 65.0)	57.5 (53.3, 63.0)	0.645
Sex	8F:6M	8F:6M	8F:6M	8F:6M	1.000
MODS	4.0 (3.0, 5.5)	6.0 (3.0, 8.0)			0.286
SOFA	4.5 (2.0, 9.3)	6.0 (4.3, 10.5)			0.204
**Comorbidities,** ***n*** **(%)**					
Hypertension	7 (50.0)	9 (64.3)			0.445
Diabetes	5 (35.7)	5 (35.7)			1.000
Chronic kidney disease	2 (14.3)	1 (7.1)			1.000
Cancer	2 (14.3)	1 (7.1)			1.000
COPD	1 (7.1)	3 (21.4)			0.596
Heart disease	2 (14.3)	2 (14.3)			1.000
Chronic heart failure	0 (0)	2 (14.3)			0.481
**Baseline labs**					
White blood count	8.5 (6.9, 16.1)	15.3 (11.1, 20.5)			0.056
Neutrophils	7.3 (5.6, 12.6)	12.2 (8.6, 15.7)			0.062
Lymphocytes	0.7 (0.6, 1.0)	1.3 (0.5, 1.8)			0.093
Platelets	206 (134, 294)	202 (164, 260)			0.872
Hemoglobin	122 (102, 135)	124 (102, 138)			0.818
Creatinine	82 (58, 187)	75 (54, 113)			0.448
**Chest X-ray,** ***n*** **(%)**					
Bilateral pneumonia	13 (92.9)	2 (14.3)			**<0.001**
Unilateral pneumonia	1 (7.1)	8 (57.1)			**0.013**
Interstitial infiltrates	0 (0)	1 (7.1)			1.000
Normal	0 (0)	3 (21.4)			0.222
PaO_2_:FiO_2_ ratio	107 (66, 162)	172 (138, 312)			0.015
Lactate	1.5 (1.0, 2.0)	1.2 (0.9, 1.6)			0.233
**Sepsis diagnosis**					
Suspected	0 (0)	10 (71.4)			**<0.001**
Confirmed	14 (100)	4 (28.6)			**<0.001**
**Study interventions**					
Antibiotics	14 (100)	14 (100)			1.000
Anti-virals	3 (21.4)	2 (14.3)			1.000
Steroids	3 (21.4)	5 (35.7)			0.678
Vasoactive medications	11 (78.6)	8 (57.1)			0.420
Renal replacement	2 (14.3)	1 (7.1)			1.000
High-flow nasal cannula	8 (57.1)	1 (7.1)			0.013
Non-invasive MV	6 (42.9)	8 (57.1)			0.450
Invasive MV ventilation	10 (71.4)	11 (78.6)			1.000
**Survived**	7 (50.0)	12 (85.7)			0.103

Continuous data are presented as medians (IQRs). MODS = Multiple Organ Dysfunction Score, SOFA= Sequential Organ Failure Assessment Score, COPD = Chronic Obstructive Pulmonary Disease, and MV = mechanical ventilation. Bold p-Values highlight statistical differences between groups.

**Table 2:** Precision and clinical performance of anti-SARS-CoV-2 antibodies and immunoassays (Data provided by Diagnostics Biochem Canada Inc.; https://dbc-labs.com/; accessed on 13 May 2021).

	Anti-SARS-CoV-2 IgM	Anti-SARS-CoV-2 IgA	* Anti-SARS-CoV-2 IgG	* Anti-SARS-CoV-2 Total Ig
Catalogue #	CAN-IGM-19	CAN-IGA-19	CAN-IGG-19	CAN-IGT-19
Total CV%	11.6–14.4	10.6–16.6	8.5–13.5	7.9–15.3
Sensitivity [PPA, % (*n*)]	93.5 (31)	85.7 (91)	93.1 (116)	94.7 (114)
Specificity [NPA, % (*n*)]	98.8 (781)	99.0 (789)	98.2 (677)	99.2 (783)
Overall Agreement [OPA, % (*n*)]	98.6 (812)	97.6 (880)	97.5 (793)	98.7 (897)
Limit of Detection	1:64	1:128	1:128	1:256
ROC AUC (*p* < 0.0001)	0.987	0.993	0.992	0.988
ROC 95% Confidence Intervals	0.965, 1.009	0.988, 0.998	0.982, 1.002	0.973, 1.004
ROC Standard Error	0.0114	0.0026	0.0052	0.0078

* Health Canada COVID-19 Medical Device Authorized, CV% = Coefficient of Variation, PPA = positive percent agreement, NPA = negative percent agreement, OPA = “overall percent agreement, ROC = receiver operating characteristic, and AUC = area under the curve.

**Table 3:** Anti-SARS-CoV-2 serological responses between age- and sex-matched COVID-19 patients with either severe (ICU day-3) or mild (non-hospitalized) symptoms.

Variable	COVID-19+ Critically Ill	COVID-19+ Mildly Ill	*p*-Value
*n*	14	14	1.000
Age in years	61.0 (54.0, 67.0)	60.0 (55.8, 65.0)	0.711
Sex	8F:6M	8F:6M	1.000
**Anti-SARS-CoV-2 Ig**			
IgM	2.8 (1.5, 5.3)	2.8 (1.2, 3.3) *	0.787
IgA	4.0 (1.5, 5.8)	2.0 (1.3, 6.5)	0.582
IgG	8.1 (2.1, 14.5)	6.3 (2.5, 14.0)	0.873
Total Ig	6.6 (3.7, 10.0)	6.8 (3.9, 9.6)	0.697

Continuous data are presented as medians (IQRs). * *n* = 13, with one patient value missing.

## Data Availability

The data presented in this study are available on request from the corresponding author.
